# Inhibitory Potentiality of Secondary Metabolites Extracted from Marine Fungus Target on Avian Influenza Virus-A Subtype H5N8 (Neuraminidase) and H5N1 (Nucleoprotein): A Rational Virtual Screening

**DOI:** 10.1016/j.vas.2022.100231

**Published:** 2022-01-06

**Authors:** Md. Mukthar Mia, Mahamudul Hasan, Muhammad Milon Miah, Mohammad Abdus Shahid Hossain, Senior Executive (Veterinary Technical Services) Md. Shariful Islam, Veterinary Consultant (Public Health) Rifat Noor Shanta

**Affiliations:** a Department of Poultry Science, Faculty of Veterinary, Animal and Biomedical Sciences, Sylhet Agricultural University, Sylhet-3100, Bangladesh; b Faculty of Veterinary, Animal and Biomedical Sciences, Sylhet Agricultural University, Sylhet-3100, Bangladesh; c Veterinary surgeon, Upazila Livestock Office, Madhabpur, Habiganj. Department of Livestock Services (DLS), Bangladesh, Dhaka; d Upazila Livestock Officer Bishwanath, Sylhet. Department of Livestock Services (DLS), Bangladesh, Dhaka; e Eskayef Pharmaceuticals Ltd; f Adorsho Pranisheba, Dhaka, Bangladesh

**Keywords:** Avian influenza-A, Marine-derived compounds, Nucleoprotein, Molecular docking, Drug Prediction

## Abstract

Highly contagious avian influenza virus’ (AIV) subtypes, including H5N1 and H5N8 are considered as serious threats for poultry industry. Despite its severity, treatment and mitigation attempts are fall into baffling. Though a few approved anti-influenza medications are available, the M2 channel blockers amantadine and rimantadine, as well as the neuraminidase inhibitor oseltamivir are being less effective due to widespread drug resistance. To cope up with these circumstances, scientists have found nucleoprotein as a novice drug targeting site for H5N1. Hence, the current study used a rational screening method to find the best candidates for nucleoprotein inhibitors of H5N1 subtype and neuraminidase inhibitors for H5N8 subtype against pathogenic AIV. Finding the best candidates, molecular docking method and computational pharmacokinetics and pharmacology was developed to estimate the potential of the multi-targeting fungal-derived natural compounds for the development of drug. Chevalone E compound was found as the best inhibitor for both nucleoprotein and neuraminidase of H5N1 and H5N8 subtypes respectively, whereas, Brevione F and Brocazine-A for nucleoprotein with Penilactone-A and Aspergifuranone for neuraminidase. In case of drug prediction, the study recommends Estramustine and Iloprost against both nucleoprotein and neuraminidase. Besides these, Butorphanol, Desvenlafaxine, Zidovudine and Nadolol are the best drug candidates for nucleoprotein inhibitors, meanwhile, Sitaxentan, Ergoloid mesylate, Capecitabine and Fenoterol act as speculated candidates against neuraminidase.

## Graphical Abstract

**Figure:**
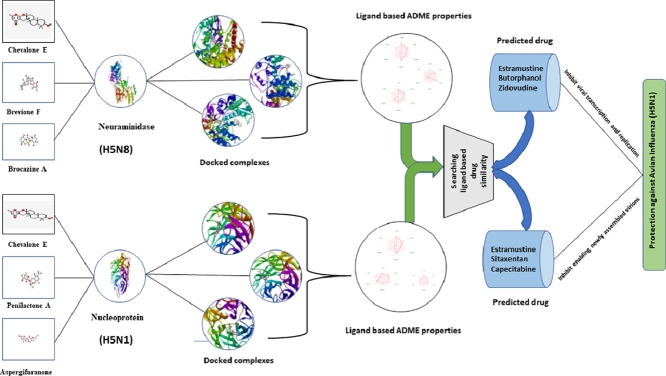

## 1 Introduction

H5 viruses of highly virulent avian influenza have pandemic potential, cause enormous economic losses, and pose a veterinary and public health risk (Hassan et al., 2020). Despite the fact that the highly pathogenic avian influenza subtype H5N1 virus was first detected in poultry in 2006, the number of human infections with the highly pathogenic avian influenza A (H5N1) virus has increased dramatically in recent decade (Peyre et al., 2009). Since 2016, the potential reintroduction of highly pathogenic avian influenza (HPAI) virus A (H5N8) from wild animals in European countries, including Austria, France, Denmark, Hungary, Germany, Poland, Netherlands, and Sweden has sparked widespread alarm throughout the world (Adlhoch et al., 2016). This reintroduction mainly aroused from wild bird, domestic migratory birds and waterfowls (FAO, 2016). In June 2017, an epidemic of the HPAI virus A (H5N8) was discovered on commercial poultry farms in South Africa's Mpumalanga Province (OIC, 2017) and the infection gradually spread throughout the country, wreaking havoc on the economy (Valley-Omar et al., 2020). Following that, H5N8 was detected from the poultry farms in Iraq (Lewis et al., 2021) and backyard chickens, ducks and geese in Russia during 2020 (Valley-Omar et al., 2020). In addition, HPAI A (H5N1) viruse caused thousands of outbreaks in poultry around the world, killing tens of millions of birds and forcing hundreds of millions more to be culled (Monamele et al., 2019). Since the first emerging in Asia in 1996, the Eurasian lineage of influenza A (H5N1) has become enzootic, especially in Southeast Asia, and has spread throughout the Asia, Europe, Africa, and North America (Tosh et al., 2016).

Pathogenic AIV possessed with 16 hemagglutinin (H1-16) and 9 neuraminidase (N1-9) subtypes, consequently, it is forecasted that AIV may prompt for the next influenza pandemic (Bialy & Shelton, 2020; Su et al., 2010). According to the World Health Organization (WHO), the majority of antiviral targets are based on M2 ion channel blockers (amantadine and rimantadine) and neuraminidase (N) inhibitors (zanamivir, oseltamivir, peramivir, and laninamivir), which are now licensed drugs for the treatment of influenza infection (Cianci et al., 2013). However, the treatment was rendered ineffective due to adamantine scarcity, lower efficacy and adverse effects of central nervous system, as well as development of rapid resistance (F G Hayden et al., 1991; Fredetick G Hayden et al., 1983). On the other hand, the neuraminidase inhibitor zanamivir has some drawbacks due to its poor oral bioavailability (Ryan et al., 1994, 1995); in addition, resistance to oseltamivir has also emerged as a result of excessive clinical use, which began in Europe in 2007 (Ciancio et al., 2009).

Previous studies reported numerous inhibitors of AIV (H5N1) hemagglutinin and neuraminidase (An et al., 2009; Ikram et al., 2015; Karthick et al., 2013); however, there is study gap in case of virtual draggability screening against nucleoprotein (NP). Thus, scientists are now hunting for influenza viral NP, which could be one of the prospective inhibitory targets for next-generation drug development (Cheng et al., 2012; Su et al., 2010) because NP plays a significant role in viral transcription and replication (Eisfeld et al., 2015). During the virus's life cycle, NP follows a substantial array of viral and host binding partners, and all its structure and function show a versatile protein with a variety of antiviral effects (Cheng et al., 2012). The acknowledged roles of NP include, but are not limited to, RNA packing organization and nuclear trafficking (Martin & Helenius, 1991). Importin, also known as karyopherin, is a cellular protein that mediates the import of viral ribonucleoprotein particles (vRNPs), which has been shown to bind to NP through the nuclear localization signal (NLS) sequence. vRNPs are connected to the inner layer of the viral membrane in the mature virion via interactions with matrix protein M1 molecules and it exposes the NLS. In the late stages of viral replication, vRNPs are brought back to the cytoplasm to be incorporated into the progeny virion, and this process is mediated by a protein assembly consisting of cellular chromosomal region maintenance 1 receptor (CRM1), M1, and vRNP transcription and replication (Jackson et al., 1982).

In contrast, new subtypes H5N8 makes the situations more badly along with existing subtypes (Normile and Enserink, 2007; Leung et al., 2012). Reassortment events between the widely distributed H5N1 virus and viruses found in waterfowl and domestic chicken have resulted in H5N8, which is causing substantial concern for the worldwide poultry sector (Bialy & Shelton, 2020). As, N is conserved in all wild-type influenza viruses, and its inhibition halts viral propagation by interfering with effective shedding and prevent cleaving sialic acid, reducing influenza virus reproduction by preventing virions from leaving host cells and spreading to new target cells. Thus, it is an attractive target for anti-influenza drug design (Ikram et al., 2015).

New medication options are desperately needed to combat the invisible enemies. Marine fungi have proven to be abundant and potential source of novel bioactive natural compounds with antiviral properties. As a result of their adaption to a particular set of circumstances, marine fungi are predicted to create unique secondary metabolites (Bhadury et al., 2006). To date, more than 275 new compounds derived from marine fungi have been found, with the number of compounds increasing all the time (Bugni & Ireland, 2004). Sargassamide, halimide, and avrainvillamide are marine fungal-derived chemicals that have shown selective suppression of cancer cell lines and in vivo action in preclinical models (P-388 lymphocytic leukemia) (http://www.cancer.ucsd.edu/summaries/wfenical.asp). The range of natural chemicals produced by marine fungi suggests that some of these substances could be used in clinical trials for developing anti-infective medications in the future. Fungal secondary metabolites with potent antiviral activity was already reported against a variety of known pathogenic viruses such as the human immunodeficiency virus, influenza virus, herpes simplex virus, hepatitis C virus, and chikungunya virus (Mia et al., 2021) as a potential drug prototypes. Therefore, the present study used a systematic screening method to find the top candidates for NP and N inhibitors as well as prediction of potential drug candidates against the pathogenic H5N8 and H5N1 subtypes. Furthermore, a computational absorption, distribution, metabolism, excretion, and toxicity (ADMET) was developed to estimate the potential of these multi-targeting fungal natural compounds for lead optimization and drug development.

## 2 Methods and materials

### 2.1 Protein dataset

PDB (Protein Data Bank) was used to find X-ray crystallographic structures of H5N8 subtypes’ neuraminidase in complex with peramivir (PDB ID: 2HTU) and H5N1 subtypes’ nucleoprotein complexed with nucleotide (PDB ID: 7DKG). The criterions were followed for selecting PDBs: (a) minimal resolution and (b) docked ligand conformation matching with the crystalline structure after redocked complexes.

### 2.2 Binding-site analysis

For N (H5N8) and NP (H5N1), high-resolution crystals with experimentally identified drug sites were predicted for docking approach. Then crystal structures were used to speculate the binding pockets of these proteins, which were evaluated using the CASTp server (Dundas et al., 2006). This server visualizes all the potential binding pockets in a solvent-accessible surface area.

### 2.3 Protein preparation

Prior to docking investigations, each protein structures were inserted into the Discovery Studio (DS) Visualizer, and the structures were refined by removing water from molecules, the original inhibitor, and the ligand. After that, missing hydrogen atoms were added and the optimization stage was performed to assure the stable conformation. Finally, the compounds were converted to PDBQT format using AutoDockTools-1.5.6 software.

### 2.4 Ligand preparation

To begin, we looked at a wide range of natural bioactive compounds derived from marine fungi that had previously been outlined in the literature (Arifeen et al., 2019; Wang et al., 2015). Following that, 162 compounds were enrolled, with ID numbers and chemical structures had taken from the PubChem database (Supplementary Table 1). Then, we refined the bioactive compounds based on the molecular weight between 350 and 500 (g/mol). Next, the biological activity of the selected compounds were predicted using the prediction server PassOnline (Filimonov et al., 2014), which envisions the biological activity spectra of compounds using the SMILES files of the structures. The likelihood of becoming active (pa) parameter was set to (pa>0,3) for a better prediction against the AIV. Finally, 41 substances were found to have antiviral properties against the AIV. Then each ligand's smile file converted to PDBQT format and inserted into AutoDockTools-1.5.6 software and set up using the prepare ligand preparation tool for docking investigation.

### 2.5 Active site prediction and molecular docking

For molecular docking analysis, AutoDock tools 1.5.6′s default approach was utilized (AutoDock 4.2 software, The Scripps Research Institute, USA). Around the active sites of AIV NP and N, as well as 7DKG and 2HTU, a grid box was formed using DS. The grid box was set to 40 × 40 × 40 points in xyz-dimension, resulting in a grid box spacing of 0.3753, and the coordinates of the x, y, and z centers of the box were fixed at 20.716826, 25.961913, and 8.969217 for NP, whereas -2.096048, 24.455746, and 13.369175 for N, respectively. For the docking simulations, included 10 Genetic Algorithm runs, the Lamarckian Genetic Algorithm with default parameters were utilized. The highest binding energy rated compounds were then investigated using the DS visualizer to investigate protein-ligand interactions.

### 2.6 Drug profile analysis of top compounds

ADME properties are the four key criterions that determine drug levels and kinetics of drug exposure to tissues within an organism and these characteristics play a big role in a drug's pharmacological activity and performance (Balani et al., 2005). The SwissADME server was used to evaluate the ADME properties of the top five metabolites (Daina et al., 2017). Besides, the blood-brain barrier (BBB) in the examined substances was calculated using the BOILED-Egg model (Daina & Zoete, 2016).

### 2.7 Toxicity, carcinogenicity, and mutagenicity prediction

The canonical SMILES of the selected compounds that showed expected antiviral activity were inserted into the pkCSM to predict toxicity, mutagenicity, and carcinogenic effects (Pires et al., 2015) and proTox-ll databases (Banerjee et al., 2018), respectively. The toxicity was predicted using the toxicity mode in the pkCSM server, and the proTox-ll software was used to assess carcinogenicity and mutagenicity. This popular server effectively predicts numerous toxicity outcomes by combining molecular similarity, fragment tendency, and fragment similarity approaches (Azim et al., 2020). Based on an analysis of two-dimensional (2D) similarity to the substances with a known median lethal dose, the server also projected oral toxicity (LD50). The list used for the prediction contains almost 38,000 different chemicals with known oral LD50 values in mice. Additionally, OSIRIS Property Explorer was used to analyze the compounds' unfavorable impacts (Drwal et al., 2014).

### 2.8 Prediction of available drug molecules from DrugBank

Based on homology screening of anticipated top drug candidates, the SwissSimilarity web tools were utilized to discover possible therapeutic compounds against AIV NP (H5N1) and N (H5N8). Using diverse methodologies such as FP2 fingerprints, electro shape, spectrophores, and align-IT (Zoete et al., 2016), the server allowed ligand-based virtual screening of several libraries of small compounds to locate authorized, investigational, or commercially available medications from the DrugBank.

## 3 Results and discussion

### 3.1 Redocking analysis against AIV-A (H5N8) neuraminidase

**Fig. 1:**
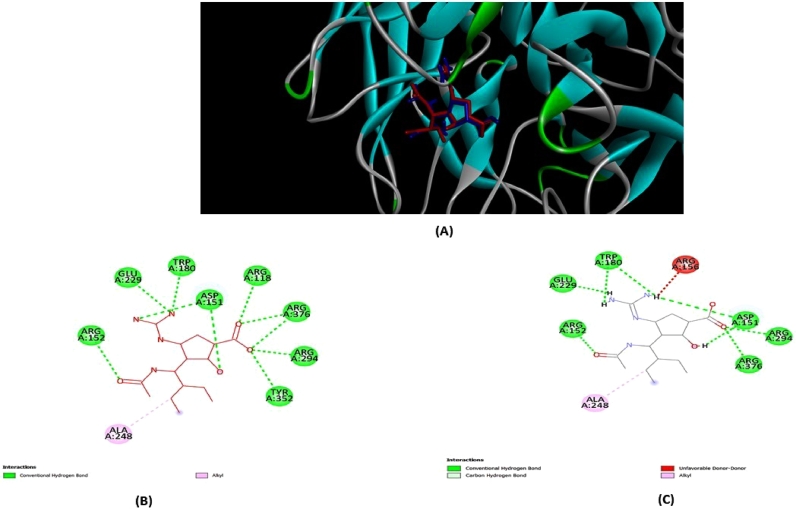
The following are the results of the validation technique used in the molecular docking study of avian influenza neuraminidase (2HTU). (A) Interactions between peramivir and 2HTU in the active site revealed overlaps between peramivir ligand from crystal (red) and AutoDock re-docking (blue). In (B) and (C), the 2D ligand-receptor interactions of peramivir from crystal and re-docking ligand using AutoDock structure with2HTU, respectively, were presented.

The crystal structures of the AIV NP (H5N1) with N (H5N8) were available in the RCSB Protein Data Bank (PDB ID: 7DKG), but established inhibitor was unavailable against 7DKG. Consequently, the NP was ignored during the screening, and only neuraminidase (PDB ID: 2HTU) was docked with the bioactive compounds. In conjunction with the inhibitor peramivir, the RCSB Protein Data Bank was used to collect AIV N (PDB ID: 2HTU). The protein structure was developed by extracting the water molecules for docking method and the inhibitor to establish a stable conformation before to the optimization stage. Then, AutoDock's basic setup was used to test the docking technique. The refined structure of the inhibitor peramivir was re-docked into the protein's original binding site. The top five genetic algorithm re-docking runs were ranked and displayed in Table 1. With a binding energy of -7.7 kcal/mol, first re-docked structure scored the best result with peramivir. The conformation, orientation, location, and interactions at the binding site of the redocked peramivir were investigated and compared to the original X-ray structure (PDB ID: 2HTU). It was revealed that peramivir was able to detect the receptor site when the redocked protein-peramivir was placed over the original X-ray structure. It's worth noting that the re-docked peramivir, as seen in Fig. 1, has adopted a subtly different amino acid structure than the one identified in the X-ray structure. The conformational variance, which could be attributed to peramivir's stable in silico structure (Rangsinth et al., 2021). Out of nine alternative models in the redocked structures, the best re-docked peramivir was shown to interact with six amino acid residues (ARG152, GLU229, TRP180, ARG294, ASP151, and ARG376). Using this docking procedure, peramivir was able to recognize its original receptor site in the X-ray structure of 2HTU.Peramivir was efficient to perceive its original receptor location in the X-ray structure of 2HTU using this docking approach. Based on the foregoing findings, the docking strategy was determined to be a suitable method for this inquiry.

**Table 1:** Hydrogen bond interaction between peramivir and 2HTU.

Fungi derived bioactive compound	2HTU		
	Binding energy (kcal/mol	Conventional Hydrogen bonding	
		Number	Amino acid interaction
X-ray structure	-	8	ARG152, GLU229, TRP180, ARG294, ASP151, ARG376, ARG118, TYR252
Re-docked structure 1	-7.7	6	ARG152, GLU229, TRP180, ARG294, ASP151, ARG376
Re-docked structure 2	-7.2	6	ARG152, GLU229, TRP180, ARG294, ASP151, ARG376
Re-docked structure 3	-6.9	4	GLU229, TRP180, ASP151, ARG376
Re-docked structure 4	-6.8	3	ARG294, GLU229, ARG156
Re-docked structure 5	-6.6	3	ARG294, GLU229, ASP151

### 3.2 Analysis of drug surface hotspot and ligand binding pocket prediction

**Fig. 2:**
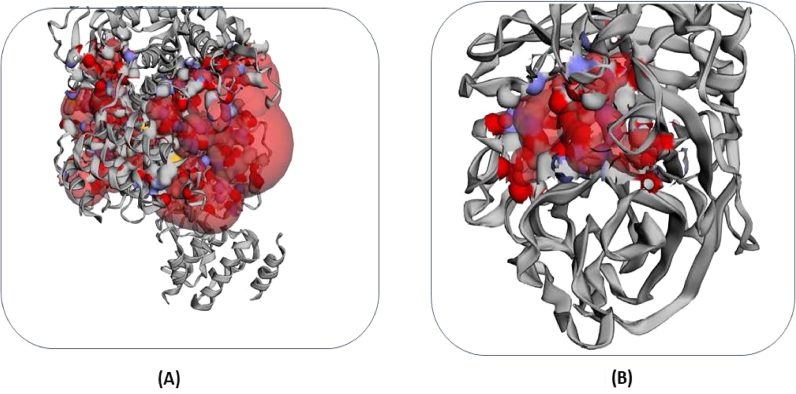
Analysis of binding pocket generated by CASTp server (A) 7DKG and (B) 2HTU.

The drug surface hotspot of the examined NP and N was investigated using the structural conformation of the docked complexes. The ligand binding pattern as well as the locations of interacting residues were studied (Table 2). The locations of amino acids 65-75, 170-175 and 494-496 were found to be crucial for NP binding interactions (7DKG). On the other hand, the amino acids from positions 224-227, 248-256, and 274-294 were crucial for N (2HTU) binding interactions. In addition, CASTp was used to confirm the binding-site residues of all three target proteins and Fig. 2 depicts the binding pocket.

**Table 2:** H-bond interactions of the top five putative marine-derived fungal compounds against the avian influenza nucleoprotein and neuraminidase inhibitor.

	Fungi derived bioactive compound	Binding energy (kcal/mol)	Conventional Hydrogen bonding	Graphical representation
			Amino acid interaction: bond length (A)	
7DKG	Chevalone E	-11.1	-	
	Brevione F	-9.0	-	
	Brocazine A	-7.8	HIS140: 2.48 ARG175: 2.93 ASP72: 2.41 ARG174: 2.16	
	Sterolic acid	-7.8	ARG75: 1.95 ARG195: 2.91 ARG150: 2.18	
	Stachybotrysin H	-7.6	ARG65: 2.44 ASN144: 2.13	
2HTU	Chevalone E	-12.7	-	
	Penilactone A	-8.5	TYR352: 2.59 ARG294: 2.28 ARG226: 2.58 GLU278: 2.34 ARG152: 2.61	
	Aspergifuranone	-7.7	ARG294: 2.78 TRP180: 2.94 GLU279: 2.27 TYR411: 2.22	
	Dehydrocurvularin	-7.7	ARG152: 2.36 AGR226: 2.61 GLU279: 2.32	
	Trichobotryside A	-7.7	GLU119: 236 ARG294: 2.78 ARG152: 1.99 TYR352: 2.15

### 3.3 Molecular docking studies

**Fig. 3:**
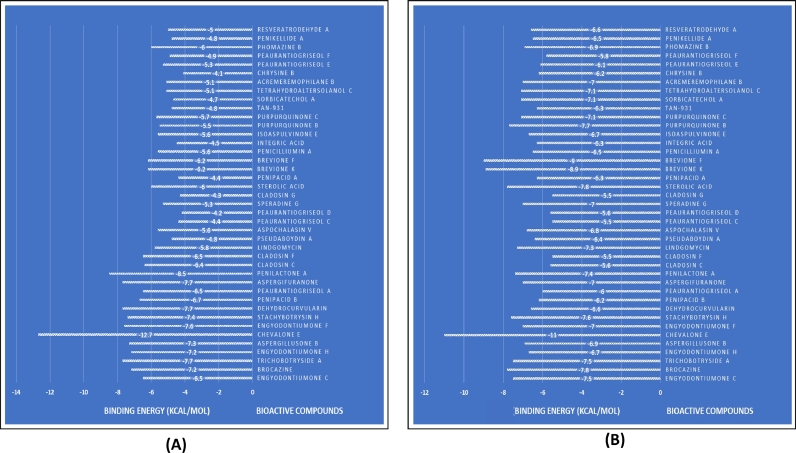
Graphical representation of receptor-ligand docked complex's binding energy. (A) 2HTU-ligands; (B) 7DKG-ligands.

The standard docking approach was used to assess candidate compounds as AIV NP (H5N1) and N (H5N8) inhibitors. Binding energy is a Gibbs free energy measure of binding affinity, and compounds with a higher negative binding energy are thought to be better (Gibbs, 1873). Previously, the in silico binding affinities of zanamivir and oseltamivir against H5N8 N were reported as -6.60 kcal/mol and -4.85 kcal/mol (Manohar, 2013), respectively, and our redocked structure of peramivir revealed -7.7 kcal/mol against N. The only licensed therapies for influenza virus infections are the N inhibitors oseltamivir, zanamivir, and peramivir (Choi et al., 2018). In contrast, there is no previous study that looks into NP; consequently, this is the most imperative study. Fig. 3 displays the docking scores of all compounds.

However, four marine fungal derived compounds, including Chevalone E (-12.7 kcal/mol), Penilactone A (-8.5 kcal/mol), Aspergifuranone (-7.7 kcal/mol), Dehydrocurvularin (-7.7 kcal/mol) had revealed the better binding affinity against N than other compounds. Meanwhile, Brocazine A (-7.8kcal/mol), Sterolic acid (-7.8 kcal/mol) showed maximum binding affinity with satisfactory hydrogen bonds against NP.

**Fig. 4:**
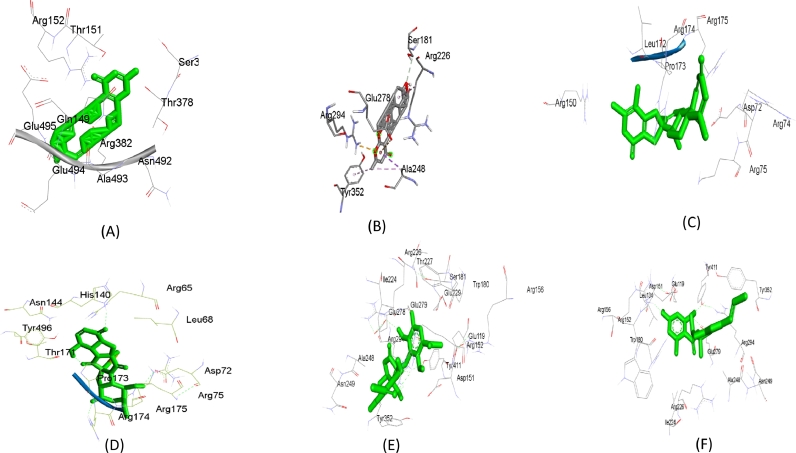
Graphical presentation of ligand interaction with protein; (A) 7DKG-Chevalone E, (B) 2HTU-Chevalone E, (C) 7DKG-Brevione F, (D) 7DKG-Brocazine A, (E) 2HTU-Penilactone A, and (F) 2HTU-Aspergifuranone.

In case of binding affinity, the complicated interactions between the avian N (H5N8) and NP (H5N1), as well as best compounds 1-5 were analyzed using DS Visualizer, and hydrogen bond analysis was performed. The amount of hydrogen bonds and its residues involved in interactions are summarized in Table 2. Possible NP inhibitor candidate molecules having drug-like characteristics, no toxicity, carcinogenicity, or mutagenicity are Sterolic acid and Brocazine A. Sterolic acid forms three hydrogen bonds at residues ARG75, ARG195, and ARG150, whereas Brocazine A makes three hydrogen bonds at residues HIS140, ARG175, ASP72, and ARG174. Penilactone A, Aspergifuranone, and Trichobotryside A have also shown that they can be utilized as N inhibitors without causing toxicity, carcinogenicity, or mutagenicity. Furthermore, at the residues ARG294, TRP180, GLU279 with TYR411, and GLU119, ARG294, ARG152, with TYR352, Aspergifuranone and Trichobotryside have four hydrogen bonds engaging with the active site of N. Interestingly, despite having a greater binding affinity, Chevalone E and Brevione F were unable to create hydrogen bonds. When compared to the X-ray structure of 2HTU-peramivir, Brocazine A with four hydrogen bonds was found to be the best inhibitor for nucleoprotein inhibitor; however, Penilactone A with five hydrogen bonds in which ARG294 and ARG152 are the common interactions revealed a potential candidate for neuraminidase inhibitor. When compared to the primal inhibitor, the studied compounds' common interactions suggest that the best marine-derived fungal compounds including, Brocazine A, Chevalone E, Brevione F, Penilactone A, and Aspergifuranone could be possible avian influenza NP and N inhibitors (ligand-receptor interactions were showed in Fig. 4).

### 3.4 Drug profile analysis of compounds

**Fig. 5:**
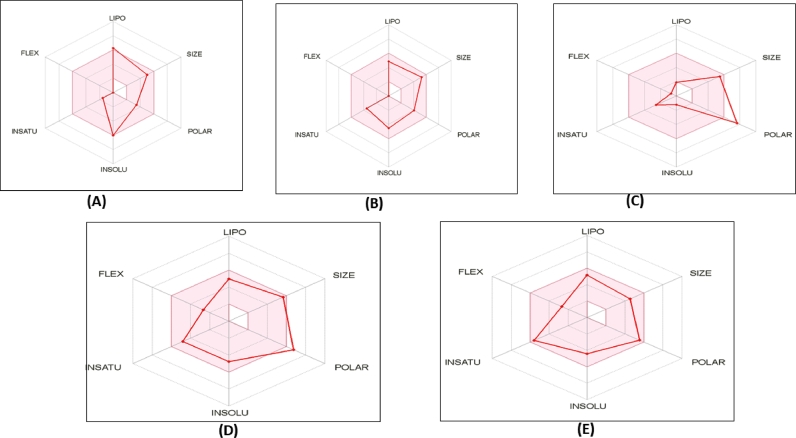
ADME analysis of top four metabolites; (A) Chevalone E, (B) Brevione F (C) Brocazine A, (D) Penilactone A, and (E) Aspergifuranone.

The ADME approach was used to analyze the drug-likeness of top five compounds found in marine-derived fungi. The prediction was made using the SwissADME database and the likeness of drug was demonstrated by Lipinski's five established rules, including the molecular weight (MW) between 350 and 500 (g/mol), the number of hydrogen bond acceptors between the range of 0 and 10, the number of hydrogen bond donors range from 0 to5, and the Log Po/w was kept between 5 and 10 (Lipinski et al., 1997), with no more than one violation allowed. Chevalone E, Brevione F, and Aspergifuranone all had higher Gastrointestinal (GI) absorption than Brocazine A and Penilactone A. Furthermore, the BOILED-Egg model was used to compute BBB penetration, which demonstrated that none of the top medication candidates tested had BBB permeation. Each contender was water soluble in varying degrees, with Brocazine A having the highest solubility (Table 3). As a result, these molecules can be utilized to mimic the effects of drugs (Fig. 5).

**Table 3:** ADME properties of top 5 bioactive compounds,

	Name	Chevalone E	Brevione F	Brocazine A	Penilactone A	Aspergifuranone
Physicochemical Properties	Formula	C26H38O4	C27H32O5	C19H20N2O7S2	C25H26O9	C20H20O7
	Molecular weight	414.58	436.54	452.5	470.47	372.37
	Num. heavy atoms	30	32	30	34	27
	Num. arom. heavy atoms	6	6	0	12	11
	Fraction Csp3	0.81	0.56	0.68	0.4	0.3
	Num. rotatable bonds	0	0	1	4	4
	Num. H-bond acceptors	4	5	7	9	7
	Num. H-bond donors	1	1	2	4	3
	Molar Refractivity	119.89	123.9	113.38	120.81	97.28
	TPSA	59.67	76.74	175.05	150.59	117.2
Lipophilicity	Log Po/w (iLOGP)	4.1	3.58	1.51	3.61	2.53
	Log Po/w (XLOGP3)	5.46	2.95	-2.16	3.2	3.56
	Log Po/w (WLOGP)	5.27	4.38	-1.69	2.62	2.64
	Log Po/w (MLOGP)	3.86	3.31	-1.61	0.86	0.71
	Log Po/w (SILICOS-IT)	5.42	4.95	-1.23	4.08	3.22
	Consensus Log Po/w	4.82	3.83	-1.04	2.87	2.53
Water Solubility	Log S (ESOL)	-6	-4.54	-1.22	-4.77	-4.43
	Solubility	4.16E-04	1.25E-02	2.73E+01	7.99E-03	1.39E-02
	Class	1.00E-06	2.86E-05	6.04E-02	1.70E-05	3.72E-05
	Log S (Ali)	Moderately soluble	Moderately soluble	Very soluble	Moderately soluble	Moderately soluble
	Solubility	-6.47	-4.22	-0.99	-6.03	-5.71
	Class	1.40E-04	2.61E-02	4.68E+01	4.35E-04	7.32E-04
	Log S (SILICOS-IT)	3.39E-07	5.97E-05	1.03E-01	9.24E-07	1.97E-06
	Solubility	Poorly soluble	Moderately soluble	Very soluble	Poorly soluble	Moderately soluble
	Class	-6	-4.54	-1.22	-4.77	-4.43
Pharmacokinetics	GI absorption	High	High	Low	Low	High
	BBB permeant	Yes	No	No	No	No
	P-gp substrate	No	Yes	Yes	Yes	No
	CYP1A2 inhibitor	Yes	No	No	No	No
	CYP2C19 inhibitor	No	No	No	No	No
	CYP2C9 inhibitor	No	No	No	Yes	Yes
	CYP2D6 inhibitor	No	No	No	No	No
	CYP3A4 inhibitor	No	Yes	No	Yes	Yes
	Log Kp (skin permeation)	-4.95	-6.87	-10.59	-6.9	-6.04
Druglikeness	Lipinski	0	0	0	0	0
	Ghose	0	0	1	0	0
	Veber	0	0	1	1	0
	Egan	0	0	1	1	0
	Muegge	1	0	2	1	0
	Bioavailability Score	0.55	0.55	0.55	0.55	0.55
Medicinal Chemistry	PAINS	0	0	0	0	0
	Brenk	0	1	1	0	0
	Leadlikeness	2	1	1	1	2
	Synthetic accessibility	5.96	6.39	6.16	4.75	4.49

### 3.5 Toxicity pattern analysis of top drug candidates

**Fig. 6:**
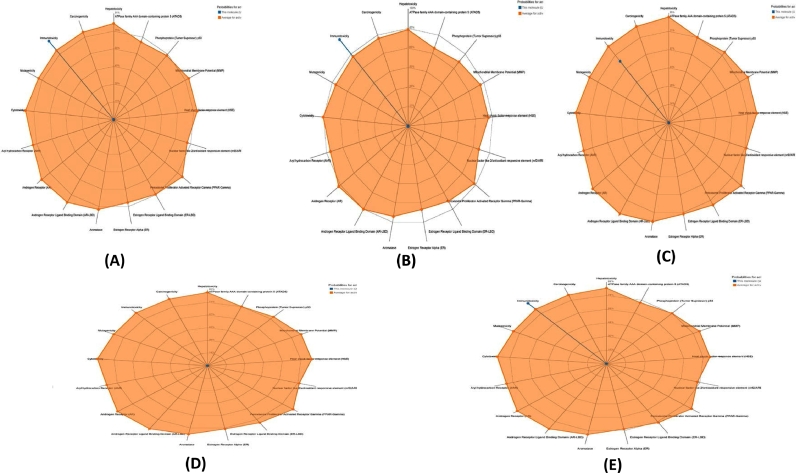
Toxicity patterns of the top four drug candidates; (A) Chevalone E, (B) Brevione F (C) Brocazine A, (D) Penilactone A, and (E) Aspergifuranone.

Prediction of various toxicity endpoints such as acute toxicity, hepatotoxicity, carcinogenicity, mutagenicity, immunotoxicity, and toxicity targets were analyzed (Table 4). Results revealed that Chevalone E, Penilactone A, and Aspergifuranone fell in the category of toxicity class 4, while Brevione F and Brocazine A showed the toxicity level 1 and 2 respectively (the lower the class the higher the toxicity). Estimated LD50 for Chevalone E, Brevione F, Brocazine A, Penilactone A, and Aspergifuranon ewere 1600mg/kg, 5mg/kg, 75mg/kg, 2500mg/kg, and 600mg/kg, respectively. The toxicity radar in Fig. 6 depicts the level of confidence in positive toxicity results when compared to the class average. There were no unfavorable effects such as tumorigenicity, mutagenicity, irritation, or reproductive consequences in any of the compounds.

**Table 4:** Toxicity, carcinogenicity and mutagenicity prediction of top 5 bioactive compounds.

Bioactive compounds name	Chevalone E	Brevione F	Brocazine A	Penilactone A	Aspergifuranone
AMES toxicity	No	No	No	No	No
hERG I inhibitor	No	No	No	No	No
hERGII inhibitor	No	No	No	No	No
Oral Rat Acute Toxicity (LD50)	2.122	2.664	3.486	2.377	2.283
Oral Rat Chronic Toxicity (LOAEL)	0.891	1.477	1.983	2.905	2.635
Hepatotoxicity	No	No	No	No	No
Skin Sensitisation	No	No	No	No	No
Minnow toxicity	1.015	0.25	5.734	1.859	1.898
Mutagenicity	Inactive (0.82)	Inactive (0.84)	Inactive (0.69)	Inactive (0.53)	Inactive (0.51)
Carcinogenicity	Inactive (0.67)	Inactive (0.53)	Inactive (0.69)	Inactive (0.60)	Inactive (0.59)
Immunotoxicity	Active (0.90)	Active (0.99)	Active (0.64)	Active (0.97)	Active (0.88)

### 3.6 Prediction of drug targets and available drug molecules from DrugBank

To predict biologically active small molecules against avian influenza-A from DrugBank, ligand-based virtual screening was used. Estramustine (DB01196) and Iloprost (DB01088), both licensed medications, were shown to be similar to Chevalone E, with prediction scores of 0.818 and 0.813, respectively. Furthermore, results revealed that Butorphanol (DB00611) and Desvenlafaxine (DB06700) are similar to Brevione F, Zidovudine (DB00495) and Nadolol (DB01203) are similar to Brocazine A, Sitaxentan (DB06268) and Ergoloid mesylate (DB01049) are similar to Penilactone A, and Capecitabine (DB01101) and Fenoterol (DB01288) (Table 5). Finally, Table 6 depicted the structure and origin of the best marine-derived chemical. The findings indicate that these could be viable therapeutic candidates for avian influenza A (H5N1 and H5N8), implying that more research is needed.

**Table 5:** Predicted drug targets for Chevalone E, Brevione F, Brocazine A, Penilactone A and Aspergifuranone.

Metabolites	Drug bank id	Name	Score	Status
Chevalone E	DB01196	Estramustine	0.818	Approved
	DB01088	Iloprost	0.813	Approved
Brevione F	DB00611	Butorphanol	0.868	Approved
	DB06700,	Desvenlafaxine	0.865	Approved
Brocazine A	DB00495	Zidovudine	0.865	Approved
	DB01203,	Nadolol	0.864	Approved
Penilactone A	DB06268	Sitaxentan	0.813	Approved
	DB01049	Ergoloid mesylate	0.808	Approved
Aspergifuranone	DB01101	Capecitabine	0.861	Approved
	DB01288	Fenoterol	0.847	Approved

**Table 6:** Best chemical compounds PubChem ID and origin.

PubChem ID	Compound Name	Structure	Origin	Reference
139587756	Chevalone E		*Aspergillus similanensis sp.*	(Prompanya et al., 2014)
44139745	Brevione F		*Penicillium sp.*	(Li et al., 2009)
118712031	Brocazine A		*Penicillium brocae MA-231*	(Meng et al., 2014)
71481658	Penilactone A		*Penicillium crustosum*	(Spence & George, 2013)
139584307	Aspergifuranone		*Aspergillus sp.*	(Liu et al., 2014)

## 4 Conclusion

The current therapeutics of the AIV is combination of zanamivir, oseltamivir, peramivir, and laninamivir drugs. Unfortunately, rapid resistance development, lesser efficacy, unfavorable effects on the central nervous system, the treatment was made ineffective. Zanamivir itself has drawbacks because of its low oral bioavailability. Consequently, scientists are looking for new drug targeting sites and NP found as the potential site for next-generation drugs development. On the other hand, marine fungus has proven to be abundant and promising source of novel bioactive natural compounds, and they may be able to inhibit the activities of viral NP and N. Furthermore, in silico ADME analysis and toxicity were used to predict these compounds' drug-like properties, toxicity, carcinogenicity, and mutagenicity. Finally, the ligand-based drug prediction was done by the SwissSimilarity server. After analyzing the findings, Chevalone E compound was found to be the best inhibitor for both NP (H5N1) and N (H5N8), whereas Brevione F and Brocazine-A were found to be the best inhibitors for nucleoprotein (eventually engage with NP, preventing bond formation with RNA and hence preventing template re-encapsidation during transcription, replication, and complementary ribonucleoprotein particles synthesis) (Tang et al., 2021), while Penilactone-A and Aspergifuranone were found to be the best blockers for neuraminidase only. We recommend Estramustine and Iloprost against both NP and N in the case of medication prediction. Apart from these, the top drug choices for nucleoprotein inhibitors are Butorphanol, Desvenlafaxine, Zidovudine, and Nadolol, whereas neuraminidase inhibitors include Sitaxentan, Ergoloid mesylate, Capecitabine, and Fenoterol. As a result, those compounds could be utilized and developed as an alternative or complementary therapy for AIV treatment. Furthermore, uncovering inhibitors with low or no toxicity will pave the door for further development as anti-AIV monotherapy or combo therapies.

## Funding information

This study received no specific support from public, private, or non-profit funding bodies.

## Ethical Statement

Hereby, I assure that for the manuscript **Virtual Screening for New Remedy from Marine Fungal-Derived Bioactive Compounds as Nucleoprotein and Neuraminidase Inhibitors of Avian Influenza A (H5N1)** following is fulfilled:

1) This material is the authors' own original work, which has not been previously published elsewhere.
2) The paper is not currently being considered for publication elsewhere.
3) The paper reflects the authors' own research and analysis in a truthful and complete manner.
4) The paper properly credits the meaningful contributions of co-authors and co-researchers.
5) The results are appropriately placed in the context of prior and existing research.
6) All sources used are properly disclosed (correct citation). Literally copying of text must be indicated as such by using quotation marks and giving proper reference.
7) All authors have been personally and actively involved in substantial work leading to the paper, and will take public responsibility for its content.

I agree with the above statements and declare that this submission follows the policies of Solid-State Ionics as outlined in the Guide for Authors and in the Ethical Statement.

## CRediT authorship contribution statement

**Md. Mukthar Mia:** Conceptualization, Methodology, Software, Visualization, Investigation, Writing – review & editing, Supervision, Validation. **Mahamudul Hasan:** Conceptualization, Methodology, Software, Writing – original draft, Visualization, Investigation, Writing – review & editing. **Muhammad Milon Miah:** Investigation, Writing – original draft. **Mohammad Abdus Shahid Hossain:** Investigation, Writing – original draft. **Senior Executive (Veterinary Technical Services) Md. Shariful Islam:** Investigation, Writing – original draft. **Veterinary Consultant (Public Health) Rifat Noor Shanta:** Investigation, Writing – original draft.

## Declaration of Competing Interest

The authors declare that they have no known competing financial interests or personal relationships that could have appeared to influence the work reported in this paper.

## Appendix Supplementary materials

Image, application 1
