# Supplementary material for: Inhibitory Potentiality of Secondary Metabolites Extracted from Marine Fungus Target on Avian Influenza Virus-A Subtype H5N8 (Neuraminidase) and H5N1 (Nucleoprotein): A Rational Virtual Screening
Source: Vet Anim Sci. 2022 Jan 6;15:100231. doi: 10.1016/j.vas.2022.100231 (PMC8760399; doi:10.1016/j.vas.2022.100231)
Supplement: Supplementary file 1 [file mmc1.docx]

**Table 1.** Marine-derive fungal bioactive compound with PubChem ID, origin and smile file.

| PubChem ID | Compound name | Origin | Smile file |
| --- | --- | --- | --- |
| 139583265 | Engyodontiumone A | Engyodontium album DFFSCS021 | COC1=C(C2=C(C=C1)OC3=CC(=CC(=C3C2=O)O)CO)C(=O)OC |
| 139587351 | Engyodontiumone B | Engyodontium album DFFSCS021 | COC(=O)C1=C(C=CC2=C1C(=O)C3=C(C=C(C=C3O2)CO)O)Cl |
| 139586117 | Engyodontiumone C | Engyodontium album DFFSCS021 | COC(=O)C1C(CCC2=C1C(=O)C3=C(C=C(C=C3O2)CO)O)O |
| 139585273 | Engyodontiumone D | Engyodontium album DFFSCS021 | CC1=CC(=C2C(=C1)OC3=C(C2=O)C(C(C(C3)O)O)C(=O)OC)O |
| 139586178 | Engyodontiumone F | Engyodontium album DFFSCS021 | COC(=O)C1C(C(CC2=C1C(=O)C3=C(C=C(C=C3O2)CO)O)O)O |
| 25762441 | Engyodontiumone H | Engyodontium album DFFSCS021 | COC(=O)C1C(C=CC2=C1C(=O)C3=C(C=C(C=C3O2)CO)O)O |
| 23872092 | Sydowinin A | Aspergillus sydowi | COC(=O)C1=C2C(=CC=C1)OC3=CC(=CC(=C3C2=O)O)CO |
| 5377796 | Pinselin | Alternaria sonchi | CC1=CC(=C2C(=C1)OC3=C(C2=O)C(=C(C=C3)O)C(=O)OC)OV |
| 45359153 | Sydowinin B | Xylaria SP. | COC(=O)C1=C(C=CC2=C1C(=O)C3=C(C=C(C=C3O2)CO)O)O |
| 53468883 | Aspergillusone B | Aspergillus sydowii PSU-F154 | COC(=O)C1(C(CCC2=C1C(=O)C3=C(C=C(C=C3O2)CO)O)O)O |
| 16204067 | Cordyol C | Cordyceps sp. | CC1=CC(=CC(=C1)OC2=CC(=CC(=C2O)O)C)O |
| 139586154 | Aspilactonol A | Aspergillus sp. | CC1C=C(C(=O)O1)CCC(C)O |
| 139586885 | Aspilactonol B | Aspergillus sp. | CC(C1C=C(C(=O)O1)C(C(C)O)O)O |
| 139586598 | Aspilactonol D | Aspergillus sp. | CC(C1C=C(C(=O)O1)C(C(C)O)OC)O |
| 38351290 | Aspilactonol | Aspergillus flocculosus | CC(CC1=CC(OC1=O)C(C)O)O |
| 139585929 | Aspyronol | Aspergillus sp. | CC1C(C=C(C(=O)O1)C(C(C)O)OC)O |
| 139583601 | Epiaspinonediol | Aspergillus sp. | CC(C=C(CO)C=CC(=O)C)O |
| 16196967 | Dihydroaspyrone | Aspergillus ochraceus | CC1C(C=C(C(=O)O1)CC(C)O)O |
| 24763079 | Aspinotriol A | Aspergillus ostianus | CC(C=CC(=CC(C)O)CO)O |
| 54697862 | Chaetoquadrin F | Aspergillus sp. | CC1=C(C=C(OC1=O)CC(C)O)O |
| 139587582 | Sorbicillamine E | Penicillium sp | CC=CC=CC(=C1C2C(C(C(C1=O)(C(=O)C2(C)O)C)C=CC)C(=O)C3=C(C4(C5C(=C(C=CC=CC)O)C(=C(C(=O)C5(OC4(C(C3=N)(C)O)O)C)C)O)C)O)O |
| 71481658 | Penilactone A | Penicillium crustosum | CC1C2(C(CC3=C(O2)C(=CC(=C3O)C(=O)C)C)(C(=O)O1)CC4=C(C(=CC(=C4O)C(=O)C)C)O)O |
| 71481659 | Penilactone B | Penicillium crustosum | CC1=CC(=C(C(=C1O)CC23CC4=C(C(=CC(=C4O)C(=O)C)C)OC2(C(OC3=O)CC(=O)O)O)O)C(=O)C |
| 139587257 | Cladosin F | Cladosporium sphaerospermum | CC(CC(CC(=N)C1=C(C(=C(C)C)NC1=O)O)O)O |
| 139583296 | Cladosin G | Cladosporium sphaerospermum | CC(CC(CC(=N)C1=C(C(=C(C)C)NC1=O)O)OC)O |
| 100925207 | Cerebroside A | Penicillium sp. | CCCCCCCCCCCCC=CC(C(=O)NC(COC1C(C(C(C(O1)CO)O)O)O)C(C=CCCC=C(C)CCCCCCCCC)O)O |
| 139587716 | Sterolic acid | Penicillium sp. | CC(C=CC(C)C(C)C(=O)O)C1CCC2C13CCC4(C2=CC(=O)C56C4(C7C(O7)C(C5O6)O)C)OC3 |
| 444679 | Ergosterol | Penicillium sp. | CC(C)C(C)C=CC(C)C1CCC2C1(CCC3C2=CC=C4C3(CCC(C4)O)C)C |
| 5351516 | Ergosterol peroxide | Penicillium sp. | CC(C)C(C)C=CC(C)C1CCC2C1(CCC3C24C=CC5(C3(CCC(C5)O)C)OO4)C |
| 10181133 | Cerevisterol | Xylaria species | CC(C)C(C)C=CC(C)C1CCC2C1(CCC3C2=CC(C4(C3(CCC(C4)O)C)O)O)C |
| 139586179 | Penipaline A | penicillium paneum | CC(=CCC1=C2C(=CC=C1)C3=C(N2)CNC(C3)C(=O)O)C |
| 139588462 | Penipaline B | penicillium paneum | CC(=CCC1=C2C(=CC=C1)C3=C(N2)C(NC(C3)C(=O)O)(C)C)C |
| 139583725 | Penipaline C | penicillium pane | CC(=CCC1=C2C(=CC=C1)C(=CN2)C=O)CO |
| 71712814 | 5-Chlorosclerotiamide | Aspergillus westerdijkiae DFFSCS013 | CC1(C=CC2=C3C(=CC(=C2O1)Cl)C4(C(C56C(C4(C)C)CC7(CCCN7C5=O)C(=O)N6)O)C(=O)N3)C |
| 71712815 | 10-Epi-sclerotiamide | Aspergillus westerdijkiae DFFSCS013 | CC1(C=CC2=C(O1)C=CC3=C2NC(=O)C34C(C56C(C4(C)C)CC7(CCCN7C5=O)C(=O)N6)O)C |
| 785 | Hydroquinone | Phialocephala sp. | C1=CC(=CC=C1O)O |
| 139585900 | Penipacid A | penicillium paneum | CC(=NCC(C)(C)O)NC1=CC=CC=C1C(=O)O |
| 139585372 | Penipacid b | penicillium paneum | CC(=NCC(C)(C)OC)NC1=CC=CC=C1C(=O)O |
| 139585062 | Penipacid C | penicillium paneum | CC(=NC1=CC=CC=C1C(=O)O)NC(=O)O |
| 139588435 | Penipacid D | penicillium paneum | CC(=NC1=CC=CC=C1C(=O)O)NC(=O)OC |
| 139583967 | Penipacid E | penicillium paneum | C1=CC=C(C(=C1)C(=O)O)NC=NC2=CC=CO2 |
| 102084345 | Oxosorbiquinol | Phialocephala sp. | CC=CC=CC(=C1C2C(C(C(C1=O)(C(=O)C2(C)O)C)C=CC)C(=O)C3=C(C(=C(C(C3=O)(C)O)O)C)O)O |
| 102084346 | Dihydrooxosorbiquinol | Phialocephala sp. | CC=CCCC(=O)C1=C(C2(C(C(C1C(C2=O)(C)O)C(=O)C3=C(C(=C(C(C3=O)(C)O)O)C)O)C=CC)C)O |
| 139585980 | Brevione I | Penicillium sp. | CC1=CC(C2C(C13CC4=C(O3)C(=C(OC4=O)C)C)(CCC5C2(C=CC(=O)C5(C)C)C)C)O |
| 139583308 | Brevione J | Penicillium sp. | CC1=CC(C2C3(CCC(=O)C(C3CCC2(C14CC5=C(O4)C(=C(OC5=O)C)C)C)(C)C)C)O |
| 139587992 | Brevione K | Penicillium sp. | CC1=CC(=O)C=CC2(C1CCC3(C2C(=O)C=C(C34CC5=C(O4)C(=C(OC5=O)C)C)C)C)C |
| 44139745 | Brevione F | Penicillium sp. | CC1=CC(C2C(C13CC4=C(O3)C(=C(OC4=O)C)C)(CCC5C2(C=CC(=O)C=C5C)C)C)O |
| 44820524 | Trichoderone | Trichoderma sp. | CCC1=CC(=O)C(C1O)O |
| 10272 | Aspergillic acid | Aspergillus sp. | CCC(C)C1=CN=C(C(=O)N1O)CC(C)C |
| 5378293 | Xantocillin | Penicillium commune SD-118 | [C-]#[N+]C(=CC1=CC=C(C=C1)O)C(=CC2=CC=C(C=C2)O)[N+]#[C-] |
| 139586152 | Penicilliumin A | Penicillium sp. F00120 | CC1(CCCC2(C1CCC(=C)C2CC3(CC(=O)C(=CC3=O)CO)O)C)C |
| 101802788 | Oxisterigmatocystin A | Aspergillus versicolor | COC1CC2C(O1)OC3=CC(=C4C(=C23)OC5=C(C=CC(=C5C4=O)O)OC)OC |
| 90670406 | Spiromastix sp. | Spiromastix sp. | CCCC1=C2C(=CC(=C1)O)OC3=C(C=C(C=C3OC2=O)O)CCC |
| 23396613 | Ascomycota sp. | Diorcinol | CC1=CC(=CC(=C1)OC2=CC(=CC(=C2)O)C)O |
| 49831334 | Aspergillus versicolor | Brevianamide Q | CC(C)(C=C)C1=C(C2=CC=CC=C2N1)C=C3C(=O)N4CCCC4(C(=O)N3)O |
| 11687274 | Chromocleista sp. | p-Hydroxyphenopyrrozin | C1CC2C(=C(C(=O)N2C1)O)C3=CC=C(C=C3)O |
| 15200541 | Aspergillus sp | Phenol A acid | CC1=C(C(=C(C=C1C(C)C(C)O)O)C(=O)O)O |
| 11682371 | Penicillium citrinum | Penicitrinone A | CC1C(OC2=C3C1=C(C(=O)C=C3OC4=C5C(=C(C(=C24)O)C)C(C(O5)C)C)C)C |
| 25064137 | Aureonitol | Chaetomium coarctatum | CC=CC=CC1COC(C1O)C=CC=C |
| 139583964 | Stachybogrisephenone B | Scytalidium sp. | CC1=CC(=CC(=C1C(=O)C2=C(C(=C(C=C2O)OC)Cl)O)O)OC |
| 511574 | Halovir A | Scytalidium sp. | CCCCCCCCCCCCCC(=O)NC(C)(C)C(=O)N1CC(CC1C(=O)NC(CC(C)C)C(=O)NC(C(C)C)C(=O)NC(CCC(=O)N)C(=O)NC(CC(C)C)CO)O |
| 10255275 | Arisugacin A | Penicillium sp. | CC1(C=CC(=O)C2(C1(CCC3(C2(CC4=C(O3)C=C(OC4=O)C5=CC(=C(C=C5)OC)OC)O)C)O)C)C |
| 122204305 | Isobutyrolactone II | Aspergillus terreus SCSGAF0162 | COC1(C(=C(C(=O)O1)O)C2=CC=C(C=C2)O)CC3=CC=C(C=C3)O |
| 25265784 | Aspernolide A | Aspergillus terreus SCSGAF0162 | CC1(CCC2=C(O1)C=CC(=C2)CC3(C(=C(C(=O)O3)O)C4=CC=C(C=C4)O)C(=O)OC)C |
| 71528813 | Balticolid | Ascomycetous strain 222 | CC1CC=CC(=O)CC=CC(CC(=O)O1)O |
| 54684703 | Equisetin | Fusarium heterosporum | CC=CC1C=CC2CC(CCC2C1(C)C(=C3C(=O)C(N(C3=O)C)CO)O)C |
| 54693801 | Phomasetin | Phoma sp. | CC=CC=CC1C(=CC2CC(CCC2C1(C)C(=C3C(=O)C(N(C3=O)C)CO)O)C)C |
| 44550889 | Integric acid | Xylaria sp. | CCCCC(C)C=C(C)C(=O)OC1CCC(C2(C1=CC(=O)C(C2)C(=C)C=O)C)C(=O)O |
| 71725700 | Oxoglyantrypine | Cladosporium sp | C1=CC=C2C(=C1)C(=CN2)CC3C(=O)NC(=O)C4=NC5=CC=CC=C5C(=O)N34 |
| 71725622 | Norquinadoline A | Cladosporium sp | CC1C(=O)N2C(N1)C(C3=CC=CC=C32)(CC4C(=O)NC(=C(C)C)C5=NC6=CC=CC=C6C(=O)N45)O |
| 185989 | Deoxynortryptoquivaline | Cladosporium sp | CC1C(=O)N2C(N1)C3(CC(C(=O)O3)N4C(=O)C5=CC=CC=C5N=C4C(C(C)C)OC(=O)C)C6=CC=CC=C62 |
| 71725699 | Deoxytryptoquivaline | Cladosporium sp | CC(C)C(C1=NC2=CC=CC=C2C(=O)N1C3CC4(C5NC(C(=O)N5C6=CC=CC=C64)(C)C)OC3=O)OC(=O)C |
| 108075 | Tryptoquivaline | Cladosporium sp | CC(C)C(C1=NC2=CC=CC=C2C(=O)N1C3CC4(C5N(C6=CC=CC=C64)C(=O)C(N5O)(C)C)OC3=O)OC(=O)C |
| 72547182 | Quinadoline B | Cladosporium | C1CC2C(=O)N3C(N2C1)C4(CC5C(=O)NC4C6=NC7=CC=CC=C7C(=O)N56)C8=CC=CC=C83 |
| 136845977 | Cladosin C | Cladosporium sphaerospermum 2005-01-E3 | CC(CC=CC(=N)C1=C(C(=C(C)C)NC1=O)O)O |
| 101885283 | Rubrolide S | Aspergillus terreus OUCMDZ-1925 | CC1(CCC2=C(O1)C=CC(=C2)C=C3C(=CC(=O)O3)C4=CC=C(C=C4)O)C |
| 71726177 | Asperterrestide A | A. terreus SCSGAF0162 | CCC(C)C1C(=O)N(C(C(=O)NC2=CC=CC=C2C(=O)NC(C(=O)N1)C)C(C3=CC=CC=C3)O)C |
| 71568836 | Isoaspulvinone E | Aspergillus terreus Gwq-48 | C1=CC(=CC=C1C=C2C(=C(C(=O)O2)C3=CC=C(C=C3)O)O)O |
| 53363661 | Emerimidine A | Emericella sp. (HK-ZJ) | COC1=C(C(=C2CNC(=O)C2=C1)O)OC |
| 53363662 | Emerimidine B | Emericella sp. (HK-ZJ) | COC1=C(C(=C2CNC(=O)C2=C1)OC)O |
| 54672324 | Purpurquinone B | Penicillium funiculosum No. 8974 | CC=CC1=CC2=CC(=O)C(C(=O)C2(C(O1)O)O)(C)OC(=O)C3=C(C(=C(C=C3O)O)O)C |
| 54672239 | Purpurquinone C | Penicillium funiculosum No. 8974 | CC=CC1=CC2=CC(=O)C(C(=O)C2(CO1)O)(C)OC(=O)C3=C(C=C(C=C3C)O)O |
| 130935 | TAN-931 | Penicillium funiculosum No. 8974 | C1=CC(=C(C(=C1)O)C(=O)C2=C(C=C(C=C2O)C(=O)O)C=O)O |
| 76310269 | Sorbicatechol A | Penicillium chrysogenum PJX-17 | CC=CC=CC(=C1C2CC(C(C1=O)(C(=O)C2(C)O)C)C3=CC(=C(C=C3)O)OC)O |
| 57332236 | Tetrahydroaltersolanol C | Alternaria sp. ZJ-2008003 | CC1(CC2C(CC1O)C(=O)C3=C(C2O)C=C(C=C3O)OC)O |
| 10348337 | Sansalvamide | Fusarium sp. | CC(C)CC1C(=O)NC(C(=O)NC(C(=O)OC(C(=O)NC(C(=O)N1)C(C)C)CC(C)C)CC(C)C)CC2=CC=CC=C2 |
| 136026468 | 2-(4-hydroxybenzyl) quinazolin-4(3H)-one | P. oxalicum 0312F1 | C1=CC=C2C(=C1)C(=O)NC(=N2)CC3=CC=C(C=C3)O |
| 132512004 | Coccoquinone A | Staphylotrichum coccosporum PF1460 | CC(=O)CCC(COC(=O)C)C1=C(C=C2C(=C1O)C(=O)C3=C(C2=O)C=C(C=C3O)O)O |
| 139202585 | Asperether C | Aspergillus wentii SD-310 | CC1(CCCC2=C3CCC4(C(COC4C3=C(C=C21)O)O)C)CO.CC1(CCCC2=C3CCC4(C(COC4C3=C(C=C21)O)O)C)CO |
| 132520154 | Acremeremophilane B | Acremonium sp. | CC=CCCC(=O)OC1C=CC2=CC(=O)C(CC2(C1C)C)C(=C)C(=O)O |
| 132520157 | Acremeremophilane E | Acremonium sp. | CC=CCCC(=O)OC1C=CC2=CC3(C(=C(C(=O)O3)C)CC2(C1C)C)O |
| 23259929 | Chaetoglobosin E | Chaetomium globosum | CC1CC=CC2C(C(=C(C3C2(C(=O)CCC(C(=O)C(=C1)C)O)C(=O)NC3CC4=CNC5=CC=CC=C54)C)C)O |
| 139587073 | Dichotocejpins A | Dichotomomyces cejpii FS110 | CN1C(=O)C2=CC3=CC=CC=C3N2C(=O)C1(CO)SC |
| 146684253 | Sarcopodinols A | Sarcopodium sp. FKJ-0025 | CCCCC1=C(C=C(C(=C1O)O)C(=O)CCCC(C)O)O |
| 146684254 | Sarcopodinols B | Sarcopodium sp. FKJ-0025 | CCCCCC(=O)C1=CC(=C(C(=C1O)O)CCCC)O |
| 146682580 | Stachybotrysin H | Stachybotrys chartarum | CC1CCC2C(C(C(CC2(C13CC4=C(C=C5COC(C5=C4O3)CC(=O)C)O)C)O)O)(C)C |
| 5359036 | Reticulol | Aspergillus sp | CC1=CC2=CC(=C(C(=C2C(=O)O1)O)OC)O |
| 139590233 | Khusinol B | Graphostroma sp. MCCC 3A00421 | CC1=CC2C(CCC(C2CC1O)CO)C(C)C |
| 132515917 | Chrysamides C | Penicillium chrysogenum SCSIO41001 | CC1C(OC(N1C(=O)C2(C(O2)C3=CC=C(C=C3)[N+](=O)[O-])C)C(=O)C)NC(=O)C4(C(O4)C5=CC=C(C=C5)[N+](=O)[O-])C |
| 146684275 | Chrysines B | Penicillium chrysogenum SCSIO 41001 | CCOC(=O)C1=C(C(=C(C(=C1OC2=C(C=C(C=C2OC)O)C(=O)OC)Cl)C)Cl)O |
| 21769029 | Brevianamide C | Penicillium brevicompactum DFFSCS025 | CC(C)C1CC23CCCN2C(=O)C1(NC3=O)C=C4C(=O)C5=CC=CC=C5N4 |
| 139589532 | Roquefortine J | Penicillium granulatum MCCC 3A00475 | CC(C)(C=C)C12C=C3C(=O)NC(=CC4=CN=CN4)C(=O)N3C1NC5=CC=CC=C25 |
| 139589750 | Spirograterpene A | Penicillium granulatum MCCC 3A00475 | CC1CC(C(C12CCC3=C2CC4(C3C(CC4)(C)C(=O)O)C)C)O |
| 6438143 | Dehydrocurvularin | Penicillium sp. SF-5859 | CC1CCCC=CC(=O)C2=C(CC(=O)O1)C=C(C=C2O)O |
| 132599608 | Trichobotryside A | Trichobotrys effuse DFFSCS021 | CC(CC1CCC(C=CC(=O)OC(CCC(C=CC(=O)O1)O)CC(C)O)O)O |
| 11355692 | Myrothenones A | Myrothecium sp. | C=CC1(CC(=CC1=O)NC=O)O |
| 197679 | Homothallin-II | Trichoderma viride strain H1-7 | C=CC1(CC(=CC1=O)C#N)O |
| 11298006 | Myrothenones B | Myrothecium sp. | C=CC1(CC(=CC1=O)N)O |
| 9913062 | Terpeptin | Aspergillus terreus 95F-1 | CC(C)C(C(=O)N(C)C(C(C)C)C(=O)NC=CC1=C(NC2=CC=CC=C21)CC=C(C)C)NC(=O)C |
| 5488797 | 5-methoxydihydrosterigmatocystin | Aspergillus versicolor MF359 | COC1=C2C(=C(C=C1)O)C(=O)C3=C(C=C4C(=C3O2)C5CCOC5O4)OC |
| 139587756 | Chevalone E | Aspergillus similanensis sp. | CC1=CC(=O)C2=C(O1)OC3(CCC4C5(CCC(C(C5CCC4(C3C2)C)(C)C)O)C)C |
| 101580161 | Xylarianaphthol-1 | Xylaria SP. | COC1=CC=CC2=C1C(=CC3=C2C4=C(O3)C5=C(C=CC=C5O)C=C4)O |
| 139586220 | lindgomycin | Lingomycetaceae | CC1CCC2C(C1)C=C(C(C2(C)C(=C3C(=O)C(NC3=O)CC4=CC=CC=C4)O)C(=CC(=O)O)C)C |
| 90676613 | Oxalicumone A | Penicillium oxalicum SCSIO 24-2 | CC1=CC(=C2C(=C1)OC3=C(C2=O)CC(S3)C(CC(C(=O)OC)O)(C(=O)OC)O)O |
| 158564 | Talaromycin A | Talaromyces sp | CCC1CCC2(CC(C(CO2)CO)O)OC1 |
| 139586677 | Pseudaboydin A | Pseudallescheria boydii | CC1(C2=C(C=C(C=C2)O)C(=O)O1)CCCC(C)(C)O |
| 90680629 | Isaridin G | Beauveria felina EN-135 | CC(C)CC1C(=O)NCCC(=O)OC(C(=O)N2CCCC2C(=O)NC(C(=O)N(C(C(=O)N1C)C(C)C)C)CC3=CC=C(C=C3)O)CC(C)C |
| 90680630 | Desmethylisaridin G | Beauveria felina EN-135 | CC(C)CC1C(=O)NCCC(=O)OC(C(=O)N2CCCC2C(=O)NC(C(=O)N(C(C(=O)N1)C(C)C)C)CC3=CC=C(C=C3)O)CC(C)C |
| 86765315 | Desmethylisaridin C1 | Beauveria felina EN-135 | CC(C)CC1C(=O)NCCC(=O)OC(C(=O)N2CCCC2C(=O)NC(C(=O)N(C(C(=O)N1)C(C)C)C)CC3=CC=CC=C3)CC(C)C |
| 139586377 | Iso-isariin D | Beauveria felina EN-135 | CCC(C)C1CC(=O)NCC(=O)NC(C(=O)NC(C(=O)NC(C(=O)NC(C(=O)O1)C)C)CC(C)C)C(C)C |
| 118719404 | 2E,4Z-tanzawaic acid D | Penicillium sp. (SF-6013) | CC1CC(CC2=C1C(=C(C=C2)C)C=CC=CC(=O)O)(C)O |
| 101885282 | Rubrolide R | Aspergillus terreus OUCMDZ-1925 | CC(=CCC1=C(C=CC(=C1)C=C2C(=CC(=O)O2)C3=CC=C(C=C3)O)O)C |
| 147621 | Questinol | Penicillium  sp. | COC1=CC(=CC2=C1C(=O)C3=C(C2=O)C=C(C=C3O)CO)O |
| 76900500 | Aspochalasin V | Aspergillus sp | CC1C2C(NC(=O)C23C(C=C(CCCC(=O)CC(C3=O)SC)C)C=C1C)CC(C)C |
| 102019164 | Penicilloside A | Candida albicans | CC1C(C(C(C(O1)OC2CCC3(C(C2)CCC4(C3CCC5(C4(C(CC5C(C)(O)OC(=O)C6=CC=CC=C6)O)O)C)O)C)O)OC)OC7C(C(C(C(O7)CO)O)O)O |
| 139588227 | Peaurantiogriseol A | Penicillium aurantiogriseum 328# | CC1CCC2C(C1O)C=CC(C2(C)C(=O)CCO)C |
| 139588399 | Peaurantiogriseol B | Penicillium aurantiogriseum 328# | CC1C=CC2CC(CCC2C1(C)C(=O)CCO)CO |
| 139588090 | Peaurantiogriseol C | Penicillium aurantiogriseum 328# | CC1C=CC2CC(CCC2C1(C)C(=O)CCO)(C)O |
| 139586324 | Peaurantiogriseol D | Penicillium aurantiogriseum 328# | CC1(CCC2C(C1)C=CC(C2(C)C(=O)CCO)(C)O)O |
| 139588522 | Peaurantiogriseol E | Penicillium aurantiogriseum 328# | CC12C=CC3CC(CCC3C1(C(=O)CCO2)C)CO |
| 139583908 | Peaurantiogriseol F | Penicillium aurantiogriseum 328# | CC1(C=CC2CC(CCC2C1(C)C(=O)CCO)CO)O |
| 139584307 | Aspergifuranone | Aspergillus sp. | CC=CC1=CC2=C(O1)C(=O)C(C(C2)O)(C)OC(=O)C3=C(C=C(C=C3C)O)O |
| 73891065 | Phomazine B | Phoma sp. OUCMDZ-1847vv | CSC12CC3=CC=CC(C3N1C(=O)C(NC2=O)(CC4=CC=CC=C4)SC)O |
| 118712031 | Brocazine | Penicillium brocae MA-231 | COC1CC(=O)C2CC34C(=O)N5C6C(CC5(C(=O)N3C2C1O)SS4)C(=O)C=CC6O |
| 139585942 | Penicibrocazine A | Penicillium brocae | CSC12CC3C(N1C(=O)C4CC5C(N4C2=O)C(CCC5=O)O)C(CCC3=O)O |
| 139584385 | Penicibrocazine B | Penicillium brocae | CSC12CC3=CC=CC(C3N1C(=O)C4CC5C(N4C2=O)C(CCC5=O)O)O |
| 139583112 | Penicibrocazine C | Penicillium brocae | CSC12CC3C(C=CC(C3N1C(=O)C4(CC5C(C=CC(C5N4C2=O)O)O)SC)O)O |
| 139585885 | Penicibrocazine D | Penicillium brocae | CSC12CC3C(N1C(=O)C4(CC5C(N4C2=O)C(CCC5=O)O)SC)C(CCC3=O)O |
| 139583686 | Penicibrocazine E | Penicillium brocae | CSC12CC3C(N1C(=O)C4(CC5C(N4C2=O)C(C=CC5=O)O)SC)C(CCC3=O)O |
| 102138423 | Penikellide A | Penicillium sp. MA-37 | CC1=CC(=C(C(=C1)O)OC2=C(C(=C(C=C2)C=CC(C)C)OC)C(=O)O)CO |
| 101890379 | Vaccinal A | Pestalotiopsis vaccinii | CC1=C(C=CC2=C1C=CC(=C2C=O)O)O |
| 102217969 | Pestalamine A | Pestalotiopsis vaccinii | CCCCOC(=O)C1=C(C=CC=C1N)C=CC(C(C)OC(=O)C2=CC=CO2)O |
| 139586455 | Flavipesin A | Aspergillus flavipes | CCCOC(=O)C1(C(=C(C(=O)O1)O)C2=CC=CC=C2)CC3=CC=CC=C3 |
| 139586286 | Resveratrodehyde A | Alternaria sp | C1=CC(=C(C=C1C=CC2=CC(=CC(=C2)O)O)C=O)O |
| 101905265 | Eleganketal A | Spicaria elegans KLA03 | CC1=C2COC3(C4=C(C(=C(C(=C4CO3)C)O)O)O)C(=O)C2=C(C(=C1O)O)O |
| 102126911 | Speradine G | Aspergillus oryzae | CC1(C2CC3=C4C(=CC=C3)N(C(=O)C4(C2C(=O)N1)O)C)C |
| 139584147 | Speradines H | Aspergillus oryzae | CC(=O)CC(=O)N1C(=O)C2=C(C1(C)C)C=C3C=CC=C4C3=C2C(=O)N4C |
| 11362869 | Psychrophilin | Penicillium algidum | CC(C)CC1C(=O)NC2=CC=CC=C2C(=O)N3C=C(CC(C(=O)N1)[N+](=O)[O-])C4=CC=CC=C43 |
| 139585353 | Versicamide F | Aspergillus versicolor | CC1(C=CN2C(CC3(C1=NC4=C3C=CC5=C4C=CC(O5)(C)C)O)C(=O)N6CCC(C6(C2=O)OC)O)C |
| 139585744 | Penicimutanin A | Penicillium purpurogenum | CCCCCCC(C)CC(C)C=CC(=O)NC1CC2(C3C(O3)C(C4C2O4)(CC(=O)C)O)OC1N5C6C(CC7N6C(=O)C(NC7=O)CC8=CC=CC=C8)(C9=CC=CC=C95)C(C)(C)C=C |
| 139585443 | Penicimutanin B | Penicillium purpurogenum | CCCCCCC(C)CC(C)C=CC(=O)NC1CC2(C3C(O3)C(C4C2O4)(CC(=O)C)O)OC1N5C6C(CC7N6C(=O)C(NC7=O)CC8=CC=C(C=C8)O)(C9=CC=CC=C95)C(C)(C)C=C |
| 86290883 | Cladosporin A | Cladosporium sp. | CC(=O)OC1C=CC=C2C1N3C(=O)C4(CC5=CC=CC(C5N4C(=O)C3(C2)SC)O)SC |
| 86290841 | Cladosporin B | Cladosporium sp. | CC(=O)OC1C=CC=C2C1N3C(=O)C4(CC5=CC=CC=C5N4C(=O)C3(C2)SC)SC |
| 102501980 | 14-hydroxy-cyclopeptine | Aspergillus sp. SCSIOW2 | CN1C(C(=O)NC2=CC=CC=C2C1=O)CC3=CC=C(C=C3)O |
| 132594639 | Trichobotrysin A | Trichobotrys effuse DFFSCS021 | CCC(C)C1C(=O)C(=C(C2C3C(CCCC3C=C(C2C4(C(O4)C)C)C)C)O)C(=O)N1C |
| 102132270 | Arisugacin K | Penicillium echinulatum | CC1(C(CC(=O)C2(C1(CCC3(C2CC4=C(O3)C=C(OC4=O)C5=CC=C(C=C5)OC)C)O)C)O)C |
| 76178391 | Aspulvinone O | Paecilomyces variotii | CC(=CCC1=CC(=C(C=C1O)O)C2=C(C(=CC3=CC(=C(C=C3)O)CC=C(C)C)OC2=O)O)C |
| 139585251 | Varioloid A | Paecilomyces variotii EN-291 | CC(C)C1(C2=NC3=C(C=CC=CO3)C(=O)N2C(C(=O)N1)CC4=CC=CC=C4)OC |
| 139584390 | Trichodin A | Trichoderma sp. strain MF106 | CC1CC(C2C(C1)C(OC3=C2C(=O)NC=C3C4=CC=C(C=C4)O)C)C |
| 139585402 | (+/-)-Asperlone A | Aspergillus sp. 16-5C | C1C2=C(C(=CC(=C2)O)O)C(=O)C3=C4C5=C(C(=CC=C5)O)C(=O)C=C4OC31O |
| 139587066 | Microsphaerol | Microsphaeropsis sp. | CC1=C(C(=CC=C1)Cl)OC2=C(C(=C(C(=C2C)C)OC3=C(C(=C(C(=C3)O)O)OC)Cl)Cl)C |
| 137797175 | Seimatorone | Microsphaeropsis sp. | COC1=C(C(=C2C(=O)CCC(C2=C1)O)O)C=O |
